# Contralateral repeated bout effect in the triceps surae after unilateral heel drop exercise at 2- and 7-day interbout intervals

**DOI:** 10.1007/s00421-026-06421-4

**Published:** 2026-09-08

**Authors:** Nicole S. Jones, Dean E. Mills, Patricio A. Pincheira, Ben W. Hoffman

**Affiliations:** 1 School of Health, Psychological, and Medical Sciences, University of Southern Queensland, Ipswich, QLD Australia; 2 University of Southern Queensland, Institute for Health, Springfield, QLD Australia; 3 School of Human Movement and Nutrition Sciences, University of Queensland, Brisbane, QLD Australia

**Keywords:** Contralateral protection, Plantar-flexor strength, Eccentric exercise, Exercise-induced muscle damage, Neuromuscular adaptation

## Abstract

**Purpose:**

A single bout of unaccustomed eccentric exercise can protect against subsequent strength loss and soreness, with protection sometimes transferring to the unexercised contralateral limb. The contralateral repeated bout effect (CL-RBE) may be relevant for muscle–tendon units with comparatively small fascicle strains during eccentric tasks, such as the triceps surae, but has not been established in this muscle group. We tested whether unilateral eccentric heel drops produced evidence consistent with the CL-RBE in the triceps surae and whether its expression differed after 2- and 7-day interbout intervals.

**Methods:**

Twenty-four participants completed 225 unilateral eccentric heel drops (ECC1), then repeated the task on the opposite limb (ECC2) after 2 days (2D, n=12) or 7 days (7D, n=12). Maximal voluntary isometric plantar-flexor torque (MVIC) and soreness were assessed at baseline (PRE) and post-exercise. Surface electromyography (sEMG) amplitude (root mean square, RMS) and median frequency (MDF) were quantified during each bout.

**Results:**

At 48 h, normalised MVIC torque was higher after ECC2 than ECC1 in 2D (10.8%PRE, 95% CI 3.0–18.5%PRE) and 7D (12.6%PRE, 95% CI 4.6–20.6%PRE), with no evidence that the ECC2–ECC1 torque response differed between intervals. Soreness was lower after ECC2 only in 2D (− 3.83/60, 95% CI −6.62 to −1.05). sEMG RMS and MDF showed no muscle-specific or interval-dependent bout effects.

**Conclusion:**

Unilateral eccentric heel drops produced evidence consistent with the CL-RBE in the triceps surae, expressed as reduced contralateral strength loss across 2- and 7-day intervals. Soreness attenuation was less consistent, while sEMG indices remained unchanged.

**Supplementary Information:**

The online version contains supplementary material available at https://doi.org/10.1007/s00421-026-06421-4.

## Introduction

Unaccustomed exercise, particularly involving eccentric muscle contractions, typically induces exercise-induced muscle damage (EIMD) in skeletal muscles. EIMD is commonly characterised by delayed-onset muscle soreness (DOMS), reductions in muscle force-generating capacity, and transient impairments in neuromuscular function (e.g. reduced voluntary activation and altered reflex excitability) (Clarkson and Hubal 2002). These responses can persist for days, and for some outcomes, up to two weeks (Nosaka et al. 2001), and can temporarily impair athletic performance and complicate exercise prescription in rehabilitation settings, particularly where recovery is slow or the risk of injury is elevated (e.g. athletes in heavy training, older adults, or clinical cohorts) (Stožer et al. 2020). Therefore, identifying the factors that attenuate EIMD has practical value for eccentric exercise programming and cross-education approaches when direct loading of an injured limb is constrained (Hyldahl et al. 2017).

Following an initial bout of eccentric exercise that induces EIMD, the exercised muscle adapts such that a subsequent bout with similar task characteristics (e.g. intensity, volume, and range of motion) produces a smaller damage response. This phenomenon is known as the ipsilateral repeated bout effect (IL-RBE), and its protective effect can persist for months, with the magnitude of protection influenced in part by the size of the initial muscle damage (McHugh 2003). Notably, protection can also be expressed in the homologous unexercised muscle of the contralateral limb after unilateral eccentric exercise, termed the contralateral repeated bout effect (CL-RBE) (Howatson and van Someren 2007; Chen et al. 2023). As the protected contralateral muscle has not been mechanically loaded, the CL-RBE is commonly interpreted as reflecting non-local (neural and/or systemic) processes rather than local remodelling within the protected muscle (Hyldahl et al. 2017). Accordingly, the CL-RBE provides a useful framework for investigating non-local contributions to repeated-bout protection, particularly in tasks in which local fascicle strain may be comparatively limited (Hoffman et al. 2014).

Although the CL-RBE has been observed in human skeletal muscles, its presence and magnitude appear to be influenced by muscle-specific characteristics (Henschke et al. 2022). The strongest evidence to date has been derived from upper limb models, particularly the elbow flexors, where tendon compliance is low, fascicle strains are likely high, and unaccustomed eccentric exercise induces large strength deficits (33–40%; Chen et al. 2016). In contrast, lower limb muscle groups, such as the triceps surae, often exhibit smaller decrements following eccentric exercise (commonly <20%; Hoffman et al. 2014; Pincheira et al. 2018), consistent with differences in architecture, habitual function, and mechanical behaviour of the muscle–tendon unit (Byrne et al. 2004; Ishikawa et al. 2005). This distinction is important because the magnitude of protection conferred by the CL-RBE has been shown to be ~50% of that of the IL-RBE (Chen et al. 2023). If the absolute damage response following the initial exercise bout is modest in a given muscle group, the absolute expression of the CL-RBE may also be small, which raises uncertainty about whether contralateral protection can be reliably detected in muscles that exhibit comparatively modest EIMD (Jeon et al. 2023). Together, these observations suggest that detecting the CL-RBE may be most challenging in muscle groups that show modest EIMD, making null findings difficult to interpret without a muscle-specific rationale and a time-sensitive design (Jeon et al. 2023).

The triceps surae provides an informative model to test this issue because its muscle–tendon mechanics differ meaningfully from those of the commonly used upper-limb protocols. During plantar flexor lengthening tasks, a substantial proportion of muscle-tendon unit length can be accommodated by the compliant Achilles tendon rather than large fascicle strains within the triceps surae (Lichtwark and Wilson 2007; Pincheira et al. 2018, 2023). This mechanical buffering may reduce the disruption of force-generating structures and suggests that large-scale structural disruption alone (e.g. “sarcomere popping”; Morgan 1990) may not fully account for either the acute deficits or the protective adaptation in this muscle-tendon unit, given that in vivo eccentric exercise typically produces subcellular perturbations within an intact, compliant system (Butterfield 2010; Hody et al. 2019). In this context, protection may reflect changes in neural control during task execution (e.g., altered activation strategies) alongside local tissue-level adjustments (Hyldahl et al. 2017). Therefore, if contralateral protection is present, it may coincide with detectable differences in gross sEMG amplitude or spectral behaviour during the eccentric task, even when fascicle strain is buffered by Achilles tendon compliance (Hyldahl et al. 2017; Lichtwark and Wilson 2007).

Despite the growing evidence for the CL-RBE in other muscle groups (Howatson and van Someren 2007; Xin et al. 2014; Chen et al. 2023), it remains unknown whether the triceps surae exhibits contralateral protection after unilateral eccentric loading. Establishing this is relevant not only for determining the generalisability of the CL-RBE beyond upper-limb models but also for clarifying the balance of neural versus mechanical contributions to repeated-bout protection in a muscle-tendon unit, where local strain behaviour differs from more damage-susceptible models (Hoffman et al. 2016; Pincheira et al. 2018). In addition, the time course of the CL-RBE remains incompletely characterised. While the IL-RBE can persist for months (Hody et al. 2019), the CL-RBE may be more time-sensitive if the relevant non-local influences are transient (Chen et al. 2023). Given the relatively modest EIMD typically observed after plantar-flexor eccentric exercise (Hoffman et al. 2016; Pincheira et al. 2018, 2021b), and direct evidence that the CL-RBE is smaller than the IL-RBE in elbow- and knee-flexor models (Chen et al. 2023), the detectability and persistence of a contralateral response in the triceps surae remained uncertain. We therefore selected a 7-day interbout interval because one-week repeated-bout designs have been used in plantar-flexor IL-RBE studies and lower-limb CL-RBE studies (Pincheira et al. 2018, 2021b; Chen et al. 2018). The 2-day interbout interval was selected as an early post-ECC1 comparison because elbow-flexor CL-RBE appears absent within 0.5–12 h but detectable after at least 24 h (Chen et al. 2016) and because plantar-flexor strength and neuromuscular impairments are expected to be pronounced around 48 h after heel-drop exercise (Pincheira et al. 2021b).

This study therefore tested whether a unilateral eccentric exercise bout produces evidence consistent with CL-RBE in the triceps surae. To examine whether timing modulated contralateral protection, we compared 2-day (2D) and 7-day (7D) interbout intervals. Traditional markers of EIMD (strength loss and muscle soreness) were assessed to establish the presence of contralateral protection (Chen et al. 2023). Surface electromyography (sEMG) was recorded during the eccentric task to explore whether any contralateral protection was accompanied by differences in task-level activation behaviour, indexed by RMS amplitude and median frequency (Starbuck and Eston 2012; Jeon et al. 2023). We hypothesised that prior unilateral eccentric exercise would confer protection to the contralateral triceps surae, evidenced by attenuated strength loss and reduced soreness, with a larger or more detectable effect after 2 days than after 7 days. We further hypothesised that this protection would occur without detectable changes in sEMG amplitude or spectral indices across bouts, consistent with the possibility that contralateral protection occurs without detectable changes in gross sEMG features during task execution.

## Materials and methods

### Participants

An a priori sample size calculation was performed using G*Power (V3.1.9.7; Faul et al. 2009) for the primary repeated-measures response in plantar-flexor MVIC torque. The calculation was informed by prior plantar-flexor repeated-bout findings (Pincheira et al. 2018, 2021b), with an anticipated effect size of *f* = 0.4, α = 0.05, and statistical power of 0.80 indicating a minimum total sample of 12 participants. As the repeated-bout response was examined at both 2- and 7-day interbout intervals, 12 participants were recruited to each group, giving a total sample of 24.

Twenty-four healthy adults were recruited and allocated to either the 2D or 7D group (n = 12 each), based on availability (non-random allocation). The groups were similar in age, anthropometrics, and PRE-ECC1 plantar flexor MVIC torque with identical sex distribution (eight females and four males per group) (Table 1).

**Table 1:** Participant characteristics of the 2-day (2D) and 7-day (7D) groups

Variable	2D (n = 12)	7D (n = 12)	*p*	*d*
Age (years)	33 ± 11	29 ± 11	0.475	0.30
Sex (F/M), n	8/4	8/4		
Height (m)	1.70 ± 0.10	1.70 ± 0.10	0.685	−0.17
Body mass (kg)	73 ± 11	71 ± 13	0.653	0.19
Body mass index (kg/m^2^)	25.5 ± 3.4	24.2 ± 3.9	0.403	0.35
PRE-ECC1 MVIC torque (N·m)	127 ± 47	131 ± 63	0.859	−0.07
ECC1 limb dominance (D/ND), n	7/5	6/6		
ECC1 limb side (R/L), n	5/7	8/4		

Note: Values are expressed as mean ± SD unless otherwise stated. Sex, ECC1 limb dominance, and ECC1 limb side are presented as counts. D, dominant; ND, non-dominant; R, right; L, left; MVIC, maximal voluntary isometric contraction torque; ECC1, first eccentric exercise bout. PRE-ECC1 MVIC torque was measured in the limb allocated to complete ECC1, which was counterbalanced by dominance

Eligibility was determined using the ESSA Adult Pre-Exercise Screening System (APSS, Version 2; Exercise & Sports Science Australia 2019) and a study-specific medical history questionnaire that screened for sensory disturbances, lower limb musculoskeletal injuries, fractures, surgical procedures, severe sprains or strains, and any condition that might limit maximal plantar flexor contraction. Participants were eligible if they were aged 18–55 years, recreationally active (≤ 1 h/week of structured/moderate-to-vigorous physical activity), and had not performed regular triceps surae resistance training, defined as more than two sessions per week in the previous six months. The exclusion criteria included ESSA-identified contraindications, lower limb injury or surgery within the previous 12 months, or the use of medications, supplements, or recovery interventions likely to affect neuromuscular or inflammatory responses. Participants maintained their usual sleep, diet, and physical activity, refrained from strenuous exercise for 72 h, alcohol for 24 h, and caffeine for 12 h before testing, and avoided new training or recovery strategies; compliance was confirmed verbally at each session. The menstrual cycle phase was not controlled due to the short data collection window, although this was not expected to materially affect strength or soreness outcomes in recreationally active adults (McNulty et al. 2020).

This study received approval from the University of Southern Queensland's Human Research Ethics Committee (H22REA079). All participants provided written informed consent, and the procedures conformed to the standards outlined in the Declaration of Helsinki (World Medical Association 2013) and the National Statement on Ethical Conduct in Human Research (National Health and Medical Research Council 2023).

### Study design

**Fig. 1:**
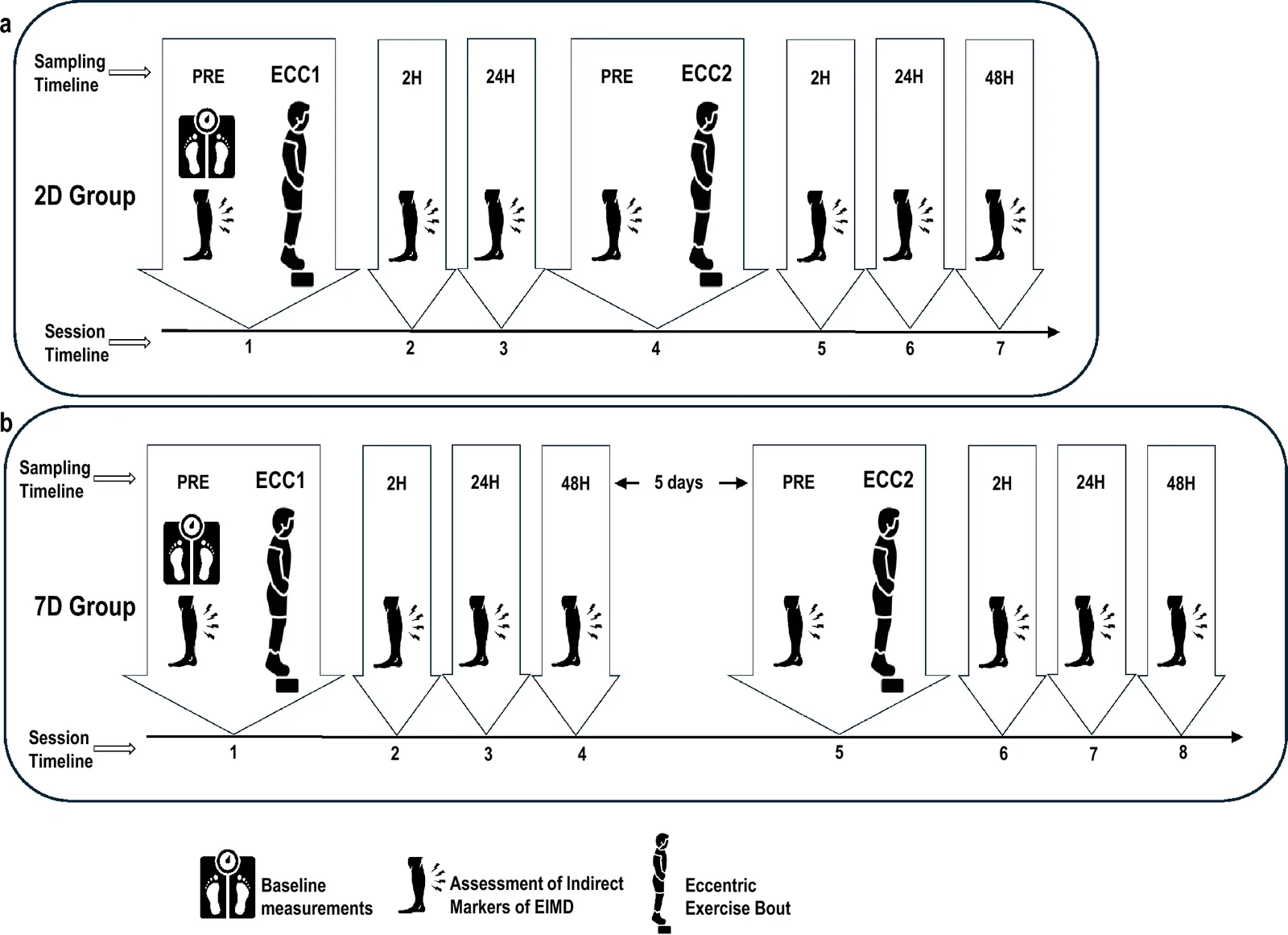
Study timeline for the 2-day (2D, panel** a**) and 7-day (7D, panel** b**) interbout interval groups. Baseline assessments (PRE) were completed before the first eccentric bout (ECC1), with follow-up assessments at 2 h (2H), 24 h (24H), and 48 h (48H) after the bout. After the assigned interbout interval, participants completed the PRE and the second eccentric bout (ECC2), followed by the same post-bout assessments. In the 2D group, the 48H post-ECC1 visit also served as the PRE-ECC2 visit; in the 7D group, PRE-ECC2 occurred five days after the 48H post-ECC1 visit. EIMD, exercise-induced muscle damage

A repeated-measures intervention design was used in which participants completed two bouts of unilateral eccentric heel drops (ECC1 and ECC2; 225 repetitions each). The limb completing ECC1 was counterbalanced by dominance, with the contralateral limb completing ECC2 after the allocated interbout interval. Assessments were performed pre-exercise (PRE) and at 2 h (2H), 24 h (24H), and 48 h (48H) after each bout (see Fig. 1). The interbout interval was 2 days (2D) or 7 days (7D), selected to sample earlier versus later recovery following eccentric exercise (Chen et al. 2018; Howatson and van Someren 2007). In the 2D group, the 48H post-ECC1 assessment coincided with the PRE-ECC2 assessment and was therefore recorded once (see Fig. 1a). In the 7D group, PRE-ECC2 occurred five days after the 48H post-ECC1 (see Fig. 1b).

For both ECC1 and ECC2, the primary outcomes, MVIC torque and composite muscle soreness, were assessed in the exercised and non-exercised limbs at PRE, 2H, 24H, and 48H to quantify indirect markers of exercise-induced muscle damage and contralateral protection. During each eccentric bout, the sEMG root mean square (RMS) amplitude and median frequency (MDF) were analysed to characterise neuromuscular activation during eccentric exercise.

Each assessment session followed a standardised sequence: soreness ratings, 10 min cycling warm-up (60 W, 60 rpm), then MVIC testing. In Session 1, the participants performed 2–3 submaximal familiarisation contractions (~ 50%, 3 s). Limb order during MVIC testing was counterbalanced, and sessions were scheduled at the same time of day (± 1 h).

All analogue signals (torque, ankle angle, vGRF, and sEMG) were recorded via a 16-channel analogue-to-digital converter (PowerLab 4/20; AD Instruments, Bella Vista, NSW, Australia) at 1000 Hz using LabChart 8. Signals were processed offline using custom LabChart 8 macros (AD Instruments, Bella Vista, Australia) and MATLAB (R2023b; MathWorks Inc., Natick, MA, USA).

### Eccentric exercise task

Each eccentric exercise bout consisted of 225 heel drops completed as 15 sets of 15 repetitions, with a 2 min rest between sets (total duration approximately 60 min). This volume was based on prior research (Pincheira et al. 2021a, b) and refined through pilot testing with the aim of inducing sufficient EIMD while remaining tolerable across two bouts. Heel drops were selected because they provide an ecologically valid, closed-chain eccentric plantar flexor task that is commonly used in calf and Achilles rehabilitation while allowing standardised loading of the triceps surae through a large ankle range of motion (Pincheira et al. 2021b). Participants wore a weighted vest equal to 10% of their body mass to increase the mechanical demand of the task. The knee was kept in full extension throughout the exercise to maximise triceps surae fascicle lengthening and the likelihood of inducing muscle damage.

Heel drops were performed while standing on an above-ground force plate (Optima BMS600900; Advanced Medical Technology Inc., Watertown, MA, USA) with the forefoot positioned at the edge of the plate and the heel free to move. From an initial position of approximately 30° plantarflexion, the participants actively lowered their heel to approximately 20° dorsiflexion at a metronome-paced descent of approximately 25°/s. A linear actuator (Heavy Duty Rod Actuator, rated force: 1.78 kN [~ 181 kg-force], stroke length: 0.23 m; Firgelli Automations Australia, Laverton, VIC, AUS) depressed one end of a wooden lever system to passively return the heel to the plantarflexed starting position, ensuring that the descent phase was active and the ascent phase was passive over a consistent six-second duty cycle. The actuator and metronome were synchronised by the investigator to maintain a uniform tempo across repetitions. The investigator observed each repetition and provided verbal coaching cues to assist participants to maintain full knee extension and complete of each heel drop through the full ankle range of motion. The participants rested in a standing position between sets.

Ankle angle (twin-axis electrogoniometer, TN1750/ST; AD Instruments, Bella Vista, NSW, AUS) and vertical ground reaction force (vGRF; force plate, BMS600900; Advanced Medical Technology Inc., Watertown, MA, USA) were recorded to quantify task characteristics (time under tension, range of motion, impulse, and estimated mechanical work) to verify comparable eccentric loading between ECC1 and ECC2 and between groups. Ankle excursion was derived from the electrogoniometer signal for each valid repetition and averaged across each exercise bout. Standardised within-group differences between ECC1 and ECC2 and associated 95% confidence intervals revealed that the exercise task was performed similarly between the bouts and groups. Calculation procedures and summary data for all task characteristics are provided in Online Resource 1.

### Maximal voluntary isometric contraction torque

MVIC testing was performed in the prone position on an isokinetic dynamometer (Biodex System 4 Pro; Biodex Medical Systems Inc., Shirley, NY, USA) with the hip and knee fully extended (Hahn et al. 2012). The ankle was secured at 3° from the maximum dorsiflexion using a heel-lift-controlled protocol developed in our laboratory. The medial malleolus was aligned with the dynamometer axis of rotation, and the foot was stabilised using a figure-eight strapping technique over 7.5 cm of high-density foam (Enduro Protect 36–130, Australian Comfort Group, Carole Park, QLD, AUS). Strap tension was applied through a ratcheted footplate to minimise heel lift and was replicated between sessions. Torque was recorded concurrently with the ankle angle and sEMG.

Both limbs were tested in a randomised order, with approximately two minutes allowed for repositioning. Participants completed three unilateral MVICs per limb, each lasting three seconds, with 90 s of rest between efforts. No visual feedback was provided so participants relied on internal cues to regulate effort. Standardised verbal encouragement was provided.

Passive plantar flexor torque was defined as the mean torque over a 500 ms window of the resting baseline immediately preceding the contraction onset. Active plantar flexor torque was calculated by subtracting passive torque from the recorded torque signal. The highest active torque value from the three trials was retained for analysis. MVIC torque was normalised to the limb-specific PRE-ECC1 value (%PRE) for further analysis.

The same prone, heel-restrained dorsiflexed testing configuration was evaluated in a separate laboratory reliability dataset of 14 healthy adults and demonstrated excellent within- and between-session reliability with low measurement error (intraclass correlation coefficient [ICC] = 0.93–0.98; coefficient of variation [CV] = 4.6–8.4%; standard error of measurement [SEM] = 2–3 N·m; 95% minimal detectable change [MDC_95_] = 5–10 N·m; see Online Resource 3).

### Muscle soreness

Muscle soreness was assessed at each time point using a numerical rating scale ranging from 0 (no discomfort) to 10 (worst soreness ever experienced). Active soreness was first assessed during three functional tasks (walking, single-leg heel raise, and step-down) to capture movement-evoked discomfort. Passive soreness was evaluated immediately afterwards, with the participants in the prone position and the knee extended. The investigator applied longitudinal thumb-depth pressure to the muscle bellies of the soleus (SOL), medial gastrocnemius (MG), and lateral gastrocnemius (LG) using a consistent palpation technique. Muscle soreness ratings from the three active tasks and the three passive palpation sites were summated to produce a composite soreness score out of 60 for each limb at each time point. This composite score, reflecting both local tenderness and movement-evoked soreness, was used in all statistical analyses and figures.

### Surface electromyography

sEMG was recorded bilaterally from the MG, LG, and SOL muscles using a wireless system (Myon 320; Myon AG, Schwarzenburg, Switzerland). The skin over each site was shaved, lightly abraded with abrasive gel (NuPrep Abrasive Skin Prep Gel; Weaver and Co., Aurora, CO, USA), and cleaned with isopropyl alcohol. Bipolar Ag/AgCl electrodes with a 20-mm interelectrode distance (Kendall 200 Series Foam Electrode, Cardinal Health, Dublin, OH, USA) were positioned according to SENIAM recommendations (Hermens et al. 2000). Electrode placement sites were marked with indelible ink at the first session and were checked and refreshed at subsequent sessions using the original skin markings where visible and the same anatomical landmarks where re-marking was required. Raw sEMG signals were recorded concurrently with torque and ankle angle. RMS amplitude and MDF were selected because they provide complementary indices of sEMG amplitude and spectral content during the task, offering a gross indication of changes in muscle activation magnitude and fatigue-related spectral behaviour across bouts (Farina et al. 2010; del Vecchio et al. 2017).

sEMG signals were processed offline using MATLAB. After removing the DC offset, the signals were band-pass filtered between 20 and 450 Hz. Trials contaminated by movement artefacts were excluded after visual inspection. Within each eccentric exercise bout, ten repetitions were analysed from Sets 3, 8, and 12 to characterise neuromuscular behaviour during the early, middle, and final stages of the task. For each contraction, the RMS amplitude and MDF were calculated. RMS amplitude was extracted from the middle 50% of the eccentric phase of each contraction, identified from the descending portion of the ankle-angle signal. RMS values were normalised to the RMS obtained from a 500-ms window centred on the peak torque during the MVIC trial immediately preceding each eccentric exercise bout (PRE-ECC1 and PRE-ECC2). MDF was derived from the power spectral density of the raw signals using Welch's method (Hann window, 512-point segments, 15% overlap) and was defined as the frequency at which the cumulative spectrum reached 50% of the total power (Enoka and Duchateau 2008). RMS (normalised to MVIC) and MDF values were averaged across the ten contractions within each set, and then across the selected sets to provide a single task-averaged value per muscle per bout for statistical analysis (set was not included as a factor).

### Statistical analyses

All statistical analyses were performed in R (Version 4.5.3; R Core Team 2026). Linear mixed-effects models were fitted using lme4 (Bates et al. 2015), with Kenward-Roger inference used for fixed effects (Kenward and Roger 1997) and implemented using lmerTest (Kuznetsova et al. 2017) and pbkrtest (Halekoh and Højsgaard 2014). Planned contrasts were estimated using emmeans (Lenth and Piaskowski 2026). Statistical significance was set at *p* < 0.05. Descriptive data are presented as mean ± SD. Between-group comparisons for baseline participant characteristics were performed using independent-samples t-tests, with standardised mean differences reported as Cohen’s d; categorical descriptors were reported as counts only. Linear mixed-effects model results are reported as *F* statistics with denominator degrees of freedom, and planned model-based contrasts are reported as mean differences with 95% confidence intervals (CI).

MVIC torque and muscle soreness were analysed using the same primary model structure. Fixed effects included interbout interval group (2D, 7D), bout (ECC1, ECC2), time (PRE, 2H, 24H, 48H), and their interactions. Participant was included as a random intercept to account for repeated observations within individuals. For all linear mixed-effects models, homoscedasticity and approximate normality of the residuals were assessed by visual inspection of residual-versus-fitted and Q-Q plots, respectively. As the composite soreness model showed upper-tail deviation and increasing residual dispersion, robustness was examined using a participant-level bootstrap sensitivity analysis on the original scale with 5000 resamples, in which participants were resampled within each interbout interval group and their complete repeated-measures profiles were retained.

For MVIC torque, planned post-exercise bout-to-bout contrasts compared ECC2 with ECC1 at 2H, 24H, and 48H within each interbout interval group. These contrasts were treated as a planned post-exercise contrast family and adjusted using the Holm method. For figure annotation, within-bout changes from baseline were assessed using post-exercise comparisons against PRE within each group and bout, with Dunnett adjustment applied because each post-exercise time point was compared with the same baseline reference. Between-bout differences are denoted by brackets with *p* values and within-bout differences from PRE denoted by † and ‡ symbols.

Absolute MVIC torque loss was calculated relative to bout-specific PRE and analysed using the same linear mixed-effects model structure. Absolute torque-loss contrasts compared ECC1 loss with ECC2 loss at 2H, 24H, and 48H within each interbout interval group, with Holm adjustment applied across the planned post-exercise contrast family. The 48H absolute torque-loss contrasts were interpreted against the conservative 10 N·m upper bound of the MDC_95_ derived from the laboratory reliability dataset reported in Online Resource 3, with differences exceeding 10 N·m considered greater than expected measurement error.

An ECC1-only model was also fitted for MVIC torque to determine whether the initial torque-loss response differed between interbout interval groups. This model included interbout interval group, time, and their interaction as fixed effects, with participant included as a random intercept. A baseline-adjusted sensitivity model was also fitted to post-exercise normalised MVIC torque, with limb-specific PRE-ECC1 absolute MVIC torque included as a covariate, to determine whether the primary interpretation was influenced by baseline plantar-flexor strength.

Missing data were minimal (< 5% of observations). One participant in the 7D group was missing the 48H post-ECC2 MVIC torque value. Linear mixed-effects models were fitted using the available observations, with no imputation applied.

Task-averaged normalised RMS amplitude and MDF were analysed separately using linear mixed-effects models. Fixed effects included interbout interval group, bout, muscle (SOL, MG, LG), and their interactions, with participant included as a random intercept. Planned bout-related contrasts were estimated from model-based marginal means, with multiplicity adjustment applied within the relevant contrast family.

## Results

### Maximal voluntary isometric contraction torque

**Fig. 2:**
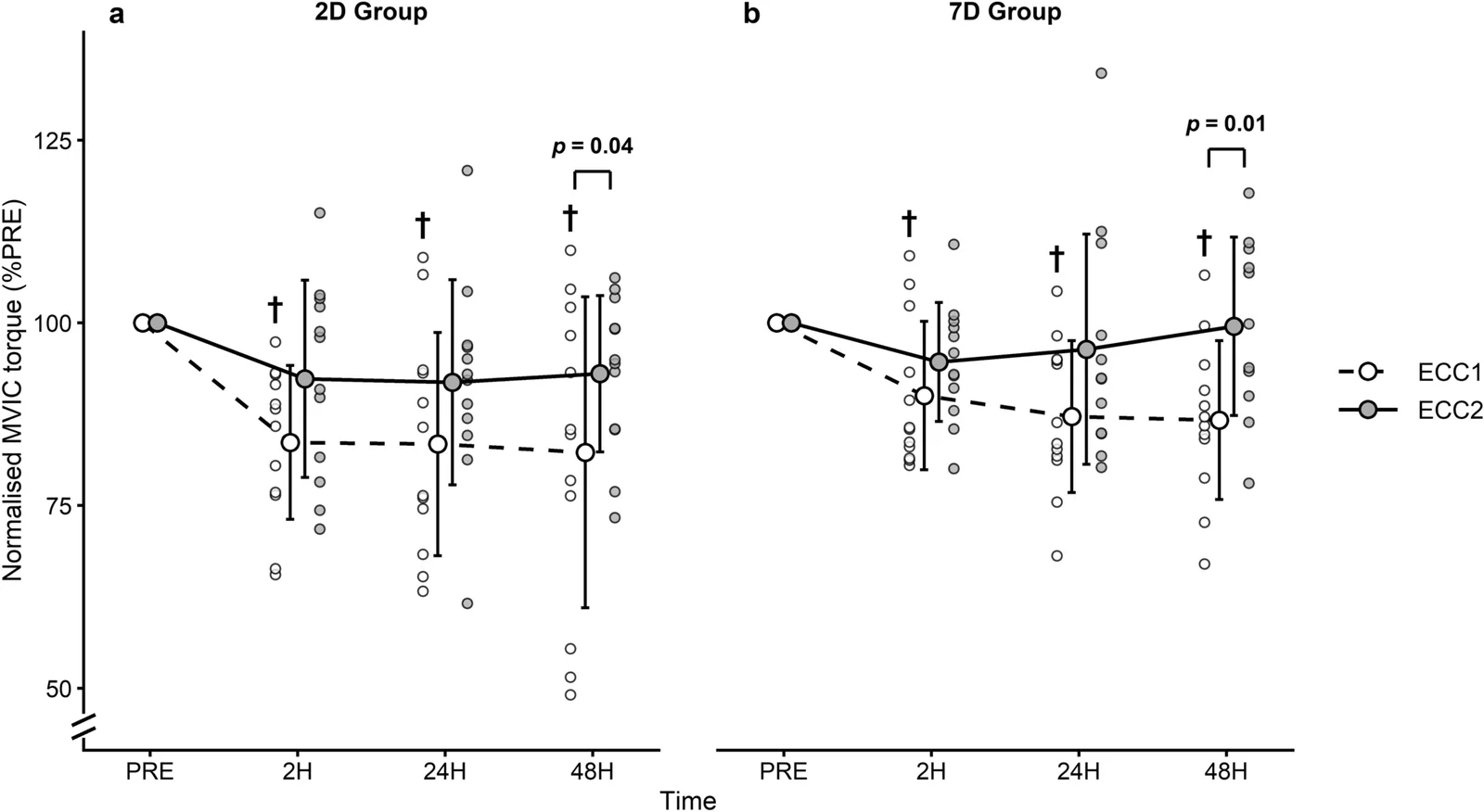
Normalised maximal voluntary isometric contraction (MVIC) torque (%PRE) following the first (ECC1, open circles and dashed lines) and second (ECC2, grey-filled circles and solid lines) eccentric exercise bouts of the triceps surae in the 2-day and 7-day interbout interval groups. Panel** a** shows the 2-day (2D) group and panel** b** shows the 7-day (7D) group. Individual participant values and group means ± SD are shown at 2 h (2H), 24 h (24H), and 48 h (48H) post-exercise. PRE is fixed at 100% by normalisation, so individual PRE values are not shown. Brackets indicate Holm-adjusted planned model-based between-bout contrasts comparing ECC2 with ECC1 at the same post-exercise time point, with adjusted* p* values shown. † Indicates Dunnett-adjusted model-based within-bout contrasts comparing ECC1 post-exercise time points with PRE (*p* < 0.05)

Figure 2 displays the normalised MVIC torque (%PRE) across time for ECC1 and ECC2 in each interbout interval group. Linear mixed-effects modelling showed significant main effects of bout (*F*_(1, 153.1)_ = 23.62, *p* < 0.001) and time (*F*_(3, 153.1)_ = 12.71, *p* < 0.001), as well as a bout × time interaction (*F*_(3, 153.1)_ = 3.16, *p* = 0.026). There was no main effect of interbout interval group (*F*_(1, 22.0)_ = 1.48, *p* = 0.237), and no group × bout (*F*_(1, 153.1)_ = 0.02, *p* = 0.894), group × time (*F*_(3, 153.1)_ = 0.72, *p* = 0.544), or group × bout × time interaction (*F*_(3, 153.1)_ = 0.22, *p* = 0.885). Thus, there was no evidence that the bout-to-bout torque response differed between the 2D and 7D groups.

Planned post-exercise bout-to-bout contrasts showed that normalised MVIC torque was higher following ECC2 than ECC1 at 48H in both interbout interval groups. In the 2D group, the model-estimated ECC2 minus ECC1 difference at 48H was 10.8%PRE (95% CI 3.0 to 18.5%PRE, *p* = 0.035). In the 7D group, the corresponding difference was 12.6%PRE (95% CI 4.6 to 20.6%PRE, *p* = 0.013). The 2H and 24H contrasts were in the same direction but were not statistically significant. The baseline-adjusted MVIC sensitivity model produced the same interpretation as the primary model, with the 48H ECC2–ECC1 torque differences remaining evident after adjustment for limb-specific PRE-ECC1 absolute MVIC torque.

Within-bout contrasts relative to PRE showed that MVIC torque was lower than PRE at 2H, 24H, and 48H after ECC1 in both the 2D group (all *p* < 0.001) and the 7D group (all *p* ≤ 0.035). In contrast, MVIC torque did not differ from PRE at any post-exercise time point after ECC2 in either the 2D group (all *p* ≥ 0.107) or the 7D group (all *p* ≥ 0.392). The corresponding absolute torque-loss contrasts at 48H showed a model-estimated attenuation after ECC2 of 16.7 N·m in the 2D group (95% CI 6.0 to 27.4, *p* = 0.015) and 15.8 N·m in the 7D group (95% CI 4.9 to 26.8, *p* = 0.025), exceeding the conservative 10 N·m upper bound of the MDC_95_ for this testing configuration (see Online Resource 3). At the individual level, 7 out of 12 participants in the 2D group and 5 out of 11 participants in the 7D group had attenuated torque loss between bouts of greater than 10 N·m. When ECC1 was analysed separately, MVIC torque decreased over time (F_(3, 66.0)_ = 14.80, *p* < 0.001), with no interbout interval group × time interaction (F_(3, 66.0)_ = 0.50, *p* = 0.685), indicating that the initial torque-loss response was similar between 2 and 7D groups.

### Muscle soreness

**Fig. 3:**
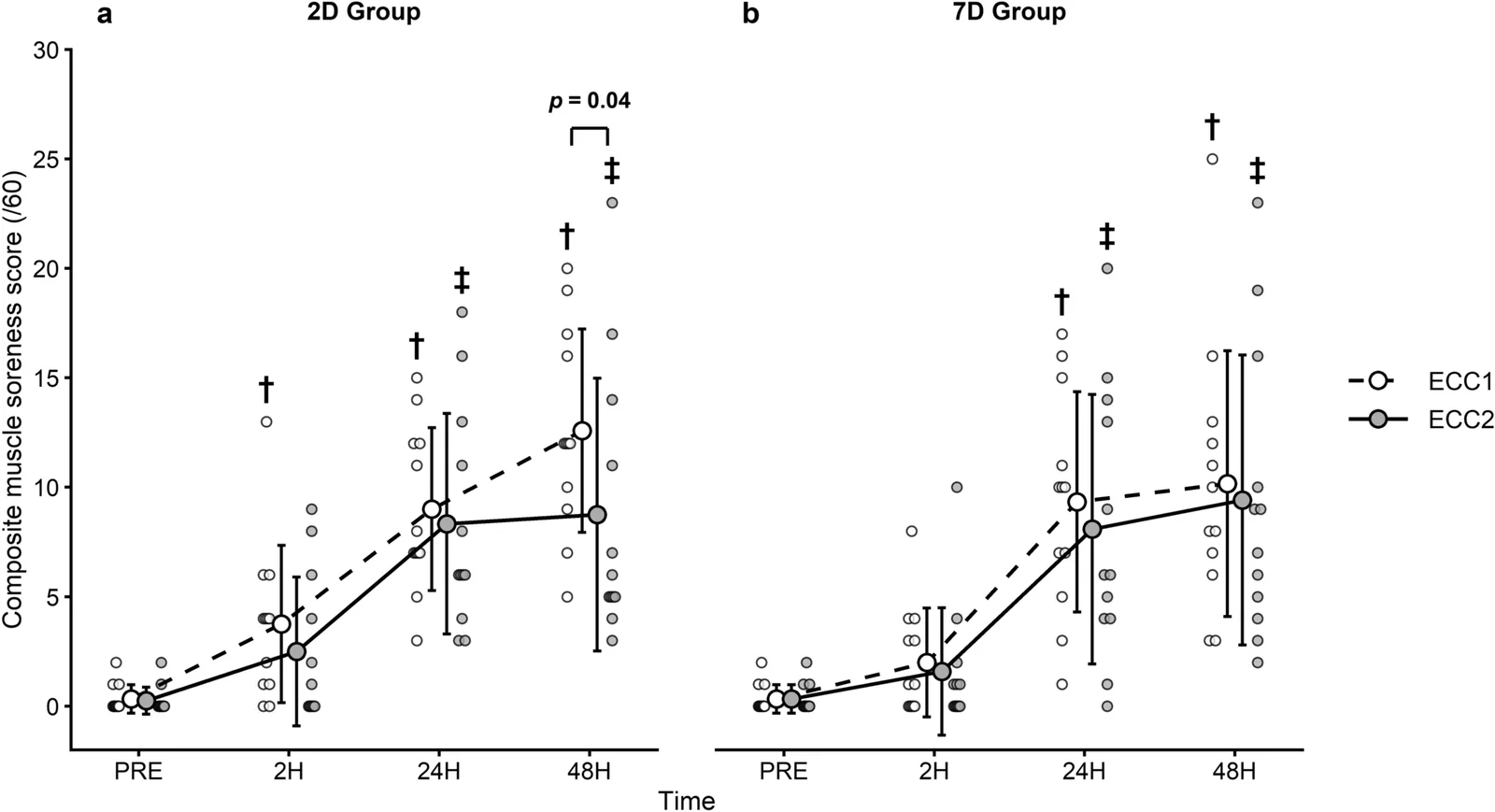
Composite muscle soreness following the first (ECC1, open circles and dashed lines) and second (ECC2, grey-filled circles and solid lines) eccentric exercise bouts of the triceps surae in the 2-day and 7-day interbout interval groups. Panel** a** shows the 2-day (2D) group and panel** b** shows the 7-day (7D) group. Individual participant values and group means ± SD are shown at PRE and 2 h (2H), 24 h (24H), and 48 h (48H) post-exercise. Composite soreness scores were calculated by summing the ratings from three active tasks and three passive palpation sites, with a maximum possible score of 60. Brackets indicate Holm-adjusted planned model-based between-bout contrasts comparing ECC2 with ECC1 at the same time point, with the adjusted *p* value shown. † and ‡ Indicate Dunnett-adjusted model-based within-bout contrasts comparing post-exercise time points with PRE for ECC1 and ECC2, respectively (*p* < 0.05)

Figure 3 displays the composite muscle soreness scores over time for ECC1 and ECC2 in each interbout interval group. Linear mixed-effects modelling showed significant main effects of bout (*F*_(1, 154.0)_ = 4.29, *p* = 0.040) and time (*F*_(3, 154.0)_ = 92.27, *p* < 0.001), with no main effect of interbout interval group (*F*_(1, 22.0)_ = 0.23, *p* = 0.636). There were no group × bout (*F*_(1, 154.0)_ = 0.74, *p* = 0.392), group × time (*F*_(3, 154.0)_ = 0.48, *p* = 0.699), bout × time (*F*_(3, 154.0)_ = 0.88, *p* = 0.454), or group × bout × time interaction effects (*F*(_3, 154.0)_ = 0.64, *p* = 0.590).

The only planned post-exercise bout-to-bout contrast that reached significance was at 48H in the 2D group, where composite soreness was lower after ECC2 than ECC1 (model-estimated difference: −3.83 points, 95% CI −6.62 to −1.05, *p* = 0.044). No between-bout differences were observed at 2H or 24H in the 2D group, with ECC2–ECC1 differences of −1.25 points at 2H (95% CI −4.03 to 1.53) and −0.67 points at 24H (95% CI −3.45 to 2.12; both *p* = 1.000). In the 7D group, the ECC2–ECC1 differences were −0.42 points at 2H (95% CI −3.20 to 2.37), −1.25 points at 24H (95% CI −4.03 to 1.53), and −0.75 points at 48H (95% CI −3.53 to 2.03; all *p* = 1.000). The sensitivity analysis supported the planned contrast interpretation (2D 48H, bootstrap *p* = 0.038; all other contrasts were non-significant). Within-bout contrasts relative to PRE showed that soreness increased after ECC1 at 2H, 24H, and 48H in the 2D group, and at 24H and 48H in the 7D group. Following ECC2, soreness increased at 24H and 48H in both interbout interval groups, but not at 2H. When ECC1 was analysed separately, composite soreness increased over time (F_(3, 66.0)_ = 62.2, *p* < 0.001), with no interbout interval group × time interaction (F_(3, 66.0)_ = 1.03, *p* = 0.386). This indicates that the post-ECC1 soreness response was similar between the 2D and 7D groups.

### Surface electromyography during the eccentric task

#### RMS amplitude

**Fig. 4:**
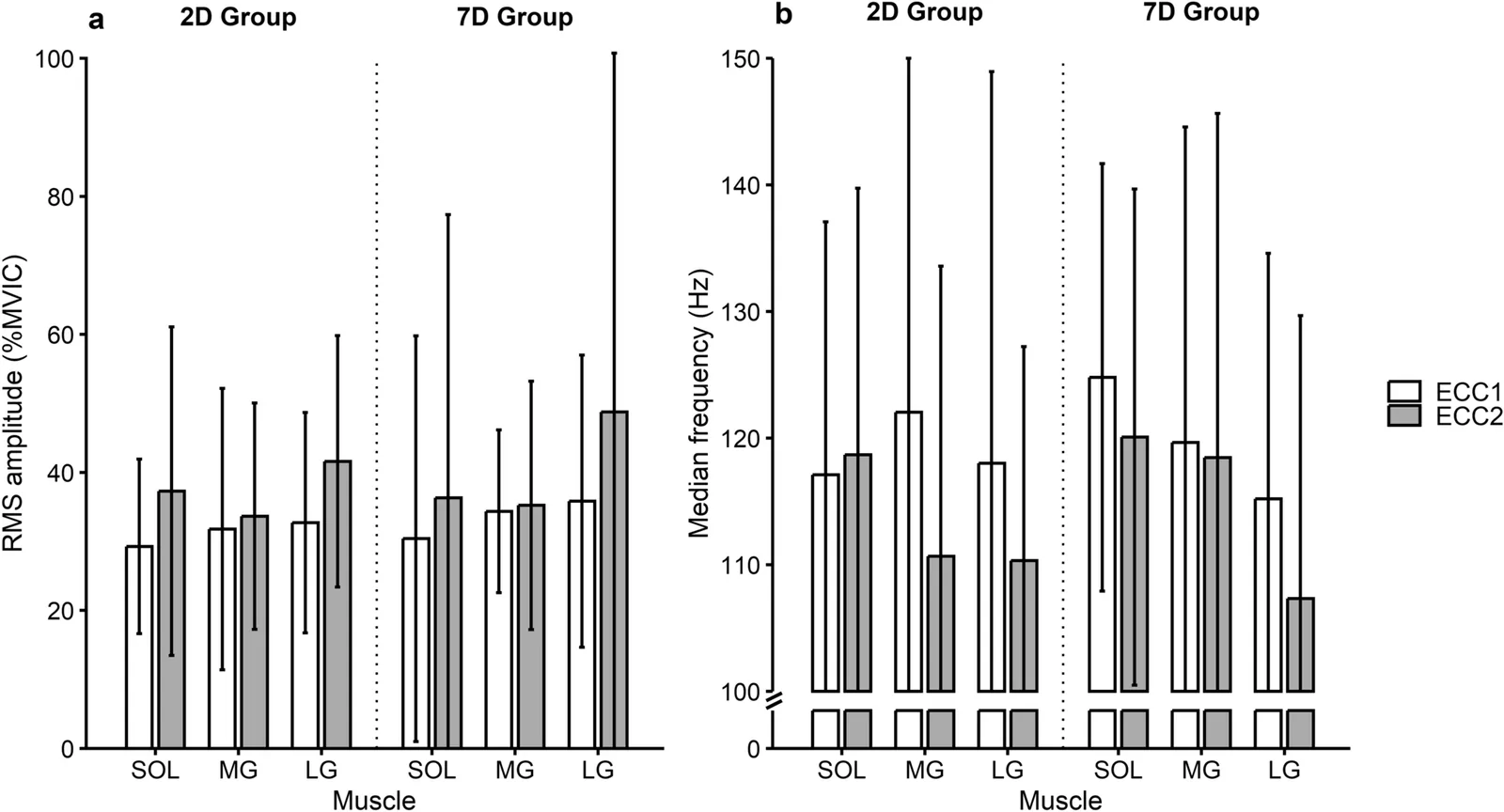
Surface electromyography (sEMG) outcomes during the eccentric exercise task. Panel **a** shows task-averaged normalised root mean square (RMS) amplitude (% maximum voluntary isometric contraction; MVIC), and panel **b** shows task-averaged median frequency (MDF; Hz), for the soleus (SOL), medial gastrocnemius (MG), and lateral gastrocnemius (LG) during the first (ECC1) and second (ECC2) eccentric exercise bouts in the 2-day (2D) and 7-day (7D) interbout interval groups. Open and grey bars represent ECC1 and ECC2, respectively. Values are presented as group means ± SD and were averaged across sets 3, 8, and 12. RMS values are displayed on the untransformed scale, although inferential analysis was performed using log-transformed values. Panel **b** uses a broken y-axis to retain the zero baseline while displaying values above 100 Hz. Individual participant data for sEMG RMS amplitude and MDF are provided in Online Resource 2

Figure 4a displays task-averaged normalised RMS amplitude (%MVIC) for ECC1 and ECC2 in each interbout interval group. Because residual diagnostics for the raw RMS model showed heteroscedasticity and tail deviation, RMS amplitude was analysed on the log scale, with raw values retained for descriptive presentation. Linear mixed-effects modelling of log-transformed RMS amplitude showed a main effect of bout (F_(1, 110.0)_ = 4.90, *p* = 0.029), but no main effects of interbout interval group (F_(1, 22.0)_ = 0.01, *p* = 0.913) or muscle (F_(2, 110.0)_ = 1.63, *p* = 0.200). There were no group × bout (F_(1, 110.0)_ = 1.39, *p* = 0.241), group × muscle (F_(2, 110.0)_ = 1.22, *p* = 0.300), bout × muscle (F_(2, 110.0)_ = 0.17, *p* = 0.846), or group × bout × muscle interaction effects (F_(2, 110.0)_ = 0.16, *p* = 0.852).

Planned bout-to-bout contrasts showed no significant ECC2–ECC1 differences for any muscle in either interbout interval group. In the 2D group, RMS amplitude for the SOL, MG, and LG was 29.27 ± 12.65, 31.78 ± 20.40, and 32.71 ± 15.96%MVIC during ECC1, and 37.28 ± 23.81, 33.66 ± 16.40, and 41.60 ± 18.23%MVIC during ECC2, respectively. In the 7D group, RMS amplitude for the SOL, MG, and LG was 30.40 ± 29.40, 34.36 ± 11.79, and 35.83 ± 21.18%MVIC during ECC1, and 36.33 ± 41.03, 35.22 ± 18.00, and 48.77 ± 51.98%MVIC during ECC2, respectively. Overall, log-transformed RMS amplitude showed a bout effect when averaged across muscles and interbout interval groups, but there was no evidence that this bout-related difference was muscle-specific or differed between the 2D and 7D groups.

#### Median frequency (MDF)

Figure 4b displays the task-averaged MDF for ECC1 and ECC2 in each interbout interval group. Linear mixed-effects modelling showed no evidence of between-bout differences in MDF for SOL, MG, or LG in either interbout interval group. In the 2D group, ECC2–ECC1 contrasts were 1.59 Hz for SOL, −11.38 Hz for MG, and −7.69 Hz for LG, with no significant differences. In the 7D group, ECC2–ECC1 contrasts were −4.71 Hz for SOL, −1.21 Hz for MG, and −7.88 Hz for LG, also with no significant differences. The same interpretation was obtained using the log-transformed MDF outcome.

In the 2D group, MDF for the SOL, MG, and LG was 117.11 ± 19.98 Hz, 122.05 ± 30.16 Hz, and 118.03 ± 30.92 Hz during ECC1, and 118.70 ± 21.05 Hz, 110.67 ± 22.92 Hz, and 110.34 ± 16.91 Hz during ECC2, respectively. In the 7D group, MDF for the SOL, MG, and LG was 124.80 ± 16.89 Hz, 119.67 ± 24.91 Hz, and 115.21 ± 19.40 Hz during ECC1, and 120.09 ± 19.59 Hz, 118.46 ± 27.20 Hz, and 107.33 ± 22.35 Hz during ECC2, respectively. Overall, there was no evidence that task-averaged MDF differed between eccentric bouts in a muscle-specific manner or that the bout-related MDF response differed between the 2D and 7D groups.

## Discussion

### The CL-RBE in the triceps surae

We examined whether a unilateral eccentric exercise bout produced evidence consistent with a contralateral repeated bout effect in the triceps surae and whether this response differed between 2-day and 7-day interbout intervals. The present findings provide the first evidence of contralateral protection in the triceps surae, expressed most consistently as preservation of normalised plantar flexor MVIC torque after ECC2. At 48H, normalised MVIC torque was better preserved after ECC2 than ECC1, with model-estimated differences of 10.8%PRE in 2D and 12.6%PRE in 7D. Soreness was less consistent, with lower composite soreness after ECC2 than ECC1 in 2D, but not 7D. sEMG RMS amplitude and MDF showed no muscle-specific between-bout differences and no interbout-interval effect. This pattern indicates a torque-dominant contralateral protective response, with less consistent soreness attenuation and no clear accompanying shift in gross task-level myoelectrical behaviour.

The magnitude of protection observed in the present study is consistent with the broader view that the CL-RBE is typically smaller than the IL-RBE, as shown in elbow-flexor, knee-extensor, and knee-flexor models (Chen et al. 2023; Jeon et al. 2023). This comparison is indirect because the present study did not include an IL-RBE condition, but previous plantar-flexor heel-drop data provide useful context. Pincheira et al. (2021b) reported 17% less strength loss and 50% less soreness at 48 h after a repeated heel-drop protocol performed 7 days after the initial bout, with participants completing ~16 ± 4 sets of 15 unilateral heel drops with 20% body mass loading. In the present study, normalised MVIC torque was better preserved after ECC2 than ECC1 by 10.8%PRE and 12.6%PRE at 48H in the 2D and 7D groups, respectively, while composite soreness was 30% lower after ECC2 in the 2D group only. These comparisons suggest that the triceps surae CL-RBE is smaller and less consistent across outcomes than the IL-RBE reported previously in the same muscle group, although this interpretation remains contextual because IL-RBE and CL-RBE were not tested under the same protocol.

Although direct numerical comparisons of the magnitude of the CL-RBE response with previous studies are limited by differences in the muscle used, contraction mode, exercise protocol and outcome scaling, the available evidence suggests that the triceps surae response may be smaller than that reported for other muscles. Compared with CL-RBE studies using a 7-day interbout interval, the present triceps surae torque response was similar in magnitude to the knee-flexor response but smaller than the elbow-flexor response, whereas soreness attenuation was smaller than in both models. In the present 7D group, normalised MVIC torque was better preserved after ECC2 than ECC1 by 12.6%PRE at 48H. In the corresponding 7-day contralateral-bout conditions reported by Chen et al. (2016, 2018), normalised torque at 48 h was ~12% greater after the contralateral bout in the knee flexors and ~24% higher in the elbow flexors. In contrast, soreness was only ~7% lower after ECC2 in the present 7D group, whereas soreness was ~59% and ~61% lower in the corresponding knee- and elbow-flexor models, respectively (Chen et al. 2016, 2018). For the shorter interbout comparison, Chen et al. (2016, 2018) did not include a 2-day condition, but their 1-day conditions provide the closest available comparator. In the present 2D group, normalised MVIC torque was higher after ECC2 than ECC1 by 10.8%PRE at 48H, which was comparable to the 1-day knee-flexor response (~ 11%) but lower than the 1-day elbow-flexor response (~ 19%). Soreness was ~30% lower after ECC2 in the present 2D group, compared with ~60% and ~69% lower soreness in the corresponding 1-day knee- and elbow-flexor models, respectively. Together, these comparisons suggest that contralateral protection can be detected in the triceps surae, but its expression may be more outcome-specific than in knee- and elbow-flexor models, with comparatively smaller attenuation of soreness. However, the present data do not establish that the overall triceps surae CL-RBE was shorter-lived than the elbow-flexor CL-RBE because torque protection remained evident at both 2 and 7 days, and intervals beyond 7 days were not examined. The less consistent soreness attenuation may instead reflect a smaller and less detectable perceptual response in a muscle group that is habitually loaded and mechanically buffered by Achilles tendon compliance, which may limit fascicle strain and the magnitude of the initial perturbation (Lichtwark and Wilson 2007; Hoffman et al. 2014; Pincheira et al. 2018, 2023).

### Interbout interval effects

A key aim of this study was to compare the magnitude of the CL-RBE across 2-day and 7-day interbout intervals. Timing effects have been tested most systematically in elbow flexor models, where the CL-RBE is not evident when the contralateral bout is performed within hours (0.5–12 h) but emerges when the interval is at least 24 h and remains detectable at 1 and 4 weeks, with weaker or absent effects at longer delays (e.g. 8 weeks) (Chen et al. 2016). Short delays have also been examined using an isometric preconditioning paradigm, in which contralateral protection across muscle-damage markers was evident at 1–2 days, whereas at 4 days attenuation was limited to soreness and no protection was apparent at 7 days, highlighting interval and protocol dependence (Chen et al. 2018a). Two-week designs are common in contralateral eccentric paradigms and have frequently demonstrated the CL-RBE across a range of damage markers, although the magnitude is typically smaller than that of ipsilateral protection (Howatson and van Someren 2007; Chen et al. 2023). Evidence for the lower limb is more limited, but knee-flexor CL-RBE was evident at 1 and 7 days but not at 28 days (Chen et al. 2018), and contralateral protection has been observed as late as ~5 weeks in a knee extensor model alongside attenuated NF-κB signalling (Xin et al. 2014). Consistent with this broader pattern, meta-analytic evidence suggests that the duration between bouts (≤ 6 weeks) moderates the contralateral effects on maximal strength (Jeon et al. 2023). In our study, the normalised MVIC torque in both groups was better preserved following ECC2 than ECC1 at 48H, with no evidence that the bout-to-bout torque response differed between the 2D and 7D groups. This indicates that contralateral torque protection can be detected in the triceps surae when a repeated bout is performed after 2 days and remains evident after a 7-day delay. The 2H and 24H contrasts were in the same direction but were not statistically significant, so the present data do not support a clear interval-dependent shift in the post-ECC2 torque time course (see Fig. 2). The interbout interval comparison suggests that the strength-preserving component of the triceps surae CL-RBE is established within the first 2 days after the initial bout and remains detectable for at least a week.

Mechanistically, the interval between 2 and 7 days may represent a period in which the acute response to ECC1 resolves while a transferable strength-preserving influence remains detectable. At 2 days, ECC2 was performed when the initial bout had still produced measurable torque loss and soreness, indicating the contralateral bout occurred before full recovery from ECC1. By 7 days, acute soreness and other short-lived post-exercise responses would be expected to have diminished, yet MVIC torque was still better preserved after ECC2. This pattern suggests that the torque component of the triceps surae CL-RBE is not dependent on the continued presence of overt soreness or acute post-exercise impairment from the first bout. It is consistent with a retained non-local adaptation involving neural, systemic or pain-related processes that is established within the first 2 days and remains functionally relevant at 7 days. Plausible pathways include bilateral changes in supraspinal or spinal control, circulating inflammatory or humoral signals, and altered pain processing (Hyldahl et al. 2017; Xin et al. 2014; Henschke et al. 2022; Locher and Koechlin 2022), although these mechanisms were not directly assessed. This interpretation aligns with contemporary accounts of repeated-bout protection as a multifactorial response involving neural, mechanical, connective-tissue, cellular, and inflammatory processes, while recognising that contralateral transfer cannot be explained by local remodelling of the protected limb before ECC2 (Paschalis et al. 2026). The less consistent soreness response suggests perceptual protection may follow a shorter or more variable time course than the maximal torque recovery. Direct assessment of the mechanisms that underpin the CL-RBE will require future studies that combine the repeated-bout design with measures of corticospinal and spinal excitability and local inflammatory markers in both limbs.

### Torque–soreness dissociation

The mismatch between torque and soreness responses has applied relevance, particularly if soreness is used to guide training progression or infer recovery after unilateral eccentric loading. Torque-based protection was evident at both interbout intervals, whereas soreness attenuation was most apparent in the 2D group. Across CL-RBE studies, soreness is typically assessed over the early post-exercise window when DOMS peaks (commonly 24–48 h), and many protocols do not extend soreness follow-up to day 7, limiting direct comparisons at later time points (Chen et al. 2016). Where 7D post-exercise data are available, soreness often returns toward baseline by the end of the first week, which reduces sensitivity to detect between-bout differences, even if earlier attenuation is present (Marathamuthu et al. 2022). Consistent with this broader pattern, the modest soreness attenuation in our 7D group likely reflects both the resolving time course of DOMS and the smaller, more variable contralateral perceptual effects reported across protocols rather than a clear absence of contralateral protection (Chen et al. 2016). This dissociation also aligns with the idea that perceived soreness integrates nociceptive sensitisation with expectation and top-down modulation, which may exhibit different persistence and decay characteristics than maximal force capacity (Locher and Koechlin 2022). In practical terms, these data caution against using soreness attenuation as the sole indicator of contralateral protection in the plantar flexors. Torque preservation appears to be a more stable marker of the CL-RBE in this muscle group under the present conditions.

### Neuromuscular activation during the task

We quantified the task-averaged sEMG RMS amplitude and MDF to examine whether contralateral protection was accompanied by detectable changes in the myoelectric amplitude or spectral behaviour during eccentric tasks. RMS is commonly used as a gross index of sEMG amplitude (overall myoelectric activity), whereas MDF characterises the distribution of spectral power and is often used to describe fatigue-related spectral shifts linked to changes in muscle fibre conduction velocity and action potential waveform (Farina et al. 2010). If contralateral protection involves altered fatigue development or peripheral myoelectrical behaviour during exercise, this could plausibly present as a bout-related shift in MDF with or without changes in RMS. However, the sEMG findings did not indicate muscle-specific between-bout differences or interbout-interval effects in RMS amplitude or MDF, suggesting that contralateral protection was not accompanied by a clear muscle-specific or interval-dependent shift in gross myoelectrical behaviour during the eccentric task. This pattern aligns with the IL-RBE research showing that protection can occur without systematic shifts in conventional bipolar sEMG features, particularly when measures are averaged across repetitions or sets and reflect gross muscle activity rather than motor unit-level behaviour (McHugh 2003).

Notably, the absence of bout-related differences in RMS and MDF does not preclude neural contributions to the CL-RBE. Bipolar sEMG provides a robust, widely used index of gross myoelectric activity during dynamic tasks. However, it does not directly resolve motor unit discharge characteristics, the distribution of recruitment across deep versus superficial motor units, or coordination across muscles within the triceps surae (Jeon et al. 2023). It also cannot localise changes occurring upstream of the muscle, such as supraspinal or spinal modulation (Henschke et al. 2022), which could influence force capacity without necessarily altering the net RMS amplitude or MDF. Accordingly, more sensitive approaches, including high-density EMG with motor unit decomposition, transcranial magnetic stimulation paradigms (e.g. paired pulse and interhemispheric inhibition), cervicomedullary stimulation, and reflex-based assessments, would be better suited to test whether the CL-RBE reflects changes in corticospinal excitability, spinal excitability, or bilateral coordination strategies (Tsuchiya et al. 2018).

### Applied implications, limitations and future directions

These findings have practical relevance for eccentric exercise prescription and lower-limb rehabilitation contexts, particularly when direct loading of one limb is temporarily limited. Eccentric heel drops are commonly used to load plantar flexors and Achilles tendon, but they can also produce transient strength loss and soreness when introduced abruptly. The present data suggest that prior eccentric loading of the opposite triceps surae may attenuate subsequent strength loss in the contralateral limb within short time frames, with torque-based protection evident after both 2-day and 7-day intervals. This may be relevant when preparing a previously unloaded, painful, or immobilised limb for progressive eccentric loading, although this application remains to be tested directly in clinical cohorts. The divergence between torque and soreness responses also has practical importance because soreness attenuation was not a consistent indicator of protection, whereas normalised MVIC torque was better preserved after ECC2. Accordingly, practitioners should not infer contralateral protection from soreness responses alone and should prioritise objective strength measures where possible. At the individual level, attenuation of torque loss exceeded the conservative 10 N·m MDC_95_ threshold in 7 out of 12 participants in the 2D group and 5 of 11 participants in the 7D group. Thus, the group-level response should not be interpreted as evidence that every participant experienced protection beyond expected measurement error, and the practical benefit of contralateral eccentric loading may vary between individuals.

Several limitations shape the interpretation of these findings. First, the fixed 225-repetition protocol may not have imposed an equivalent relative stimulus across participants. Future studies could individualise exercise volume using a predefined torque-loss criterion. Second, the study relied on functional and perceptual markers (MVIC torque and soreness) rather than direct indices of muscle structural disruption or neuromuscular adaptation, which constrains inferences about the locus of protection (peripheral tissue preservation versus centrally mediated modulation of motor output and pain perception). Third, because the design did not include a same-limb repeated bout condition, we could not directly compare the CL-RBE and the IL-RBE in the triceps surae under identical loading and assessment conditions. Accordingly, the comparison with previous plantar-flexor IL-RBE studies should be interpreted as contextual rather than definitive. The present data cannot determine whether the magnitude or the time course of contralateral protection differs from ipsilateral protection when the same heel-drop protocol is repeated by the same limb. Future studies should include both ipsilateral and contralateral repeated-bout conditions within the same protocol to quantify the relative magnitude, persistence, and outcome specificity of the triceps surae IL-RBE and CL-RBE. Fourth, participants were allocated to interbout interval groups based on availability rather than randomisation. Although the groups were similar in age, anthropometrics, sex distribution, and PRE-ECC1 MVIC torque, non-random allocation may have allowed unmeasured between-group differences. Finally, the sample size was selected for the primary MVIC torque comparison and was not designed to examine sex-specific, subgroup differences in soreness, or small muscle-specific sEMG effects. Menstrual-cycle phase was also not controlled, but the extent to which hormonal fluctuations may have influenced the present strength, soreness, or bipolar sEMG outcomes remains uncertain. The 8F:4 M distribution within each group also precluded stable sex-stratified sensitivity analyses. Future studies with larger, prospectively stratified samples may help determine whether sex, hormonal status, training history, or baseline damage susceptibility modifies the magnitude or time course of the triceps surae CL-RBE.

Future studies should directly test the neural mechanisms underpinning the CL-RBE using designs that combine the contralateral paradigm with estimates of motor unit properties or motor evoked potentials. A related question is how neural measures relate to the mechanics of the task. To address this, ultrasound tracking of fascicle and tendon behaviour during the eccentric task could quantify muscle–tendon kinematics (e.g. fascicle length change, shortening velocity, and tendon elongation) and test whether altered neural drive or motor unit coordination is associated with different mechanical strain patterns during the task, even without prior mechanical loading of the contralateral limb. Another important extension of this research is a within-protocol comparison that includes both ipsilateral and contralateral repeated bout conditions, which would quantify the relative magnitude and time course of the IL-RBE versus the CL-RBE under identical loading and assessment conditions. Finally, extending interbout intervals beyond one week would further define the persistence and decay characteristics of contralateral protection in the triceps surae and clarify whether the observed dissociation between torque and soreness is maintained as the interval increases.

## Conclusion

This study provides the first evidence consistent with a contralateral repeated bout effect in the triceps surae, expressed most robustly as the preservation of maximal strength following a repeated eccentric exercise bout performed 2 and 7 days after an initial unilateral bout. Soreness attenuation was less consistent and was observed only in the 2D group at 48H. Contralateral protection occurred without muscle-specific between-bout differences or interbout-interval effects in task-averaged sEMG RMS amplitude or MDF, suggesting that the protective effect is not explained by a clear muscle-specific or interval-dependent shift in gross myoelectric behaviour during the eccentric task. These findings broaden the physiological scope of the CL-RBE by showing that contralateral protection can be detected in a compliant, habitually loaded plantar-flexor muscle–tendon unit, while highlighting the need for targeted mechanistic studies to identify the pathways that mediate contralateral protection in the triceps surae.

## Supplementary Information

Below is the link to the electronic supplementary material.

Supplementary file1 (PDF 176 KB)

Supplementary file2 (PDF 398 KB)

Supplementary file3 (PDF 103 KB)

## Data Availability

The datasets generated and analysed during the current study are not publicly deposited because participant consent and ethics approval did not include unrestricted release through a public repository. De-identified data and analysis code may be made available from the corresponding author on reasonable request, subject to institutional ethics and data-governance requirements.

## Abbreviations

2D: 2-Day group
2H: 2 Hours post-exercise
24H: 24 Hours post-exercise
48H: 48 Hours post-exercise
7D: 7-Day group
CL-RBE: Contralateral repeated bout effect
DOMS: Delayed onset muscle soreness
ECC1: First bout of eccentric exercise
ECC2: Second bout of eccentric exercise
EIMD: Exercise-induced muscle damage
IL-RBE: Ipsilateral repeated bout effect
LG: Lateral gastrocnemius
MDF: Median frequency
MG: Medial gastrocnemius
MVIC: Maximal voluntary isometric contraction
PRE: Baseline measurement prior to exercise
RMS: Root mean square
sEMG: Surface electromyography
SOL: Soleus

## References

[CR1] Bates D, Mächler M, Bolker B, Walker S (2015) Fitting linear mixed-effects models using lme4. J Stat Softw 67:1–48. 10.18637/jss.v067.i01

[CR2] Butterfield TA (2010) Eccentric exercise in vivo: strain-induced muscle damage and adaptation in a stable system. Exerc Sport Sci Rev 38:51–60. 10.1097/JES.0b013e3181d496eb 20335736

[CR3] Byrne C, Twist C, Eston R (2004) Neuromuscular function after exercise-induced muscle damage. Sports Med 34:49–69. 10.2165/00007256-200434010-00005 14715039

[CR4] Chen TC, Chen H-L, Lin M-J, Yu HI, Nosaka K (2016) Contralateral repeated bout effect of eccentric exercise of the elbow flexors. Med Sci Sports Exerc 48:2030–2039. 10.1249/MSS.0000000000000991 27187096

[CR46] Chen TC, Lin M-J, Chen H-L, Lai J-H, Yu H-I, Nosaka K (2018a) Muscle damage protective effect by two maximal isometric contractions on maximal eccentric exercise of the elbow flexors of the contralateral arm. Scand J Med Sci Sports 28:1354–1360. 10.1111/sms.13042 29274169

[CR5] Chen TC, Lin M-J, Chen H-L, Yu H-I, Nosaka K (2018b) Contralateral repeated bout effect of the knee flexors. Med Sci Sports Exerc 50:542–550. 10.1249/MSS.0000000000001470 29077637

[CR6] Chen TC, Chen H, Tseng W, Chou T, Tu J, Parcell AC, Nosaka K (2023) Contralateral versus ipsilateral protective effect against muscle damage of the elbow flexors and knee extensors induced by maximal eccentric exercise. Scand J Med Sci Sports 33:2548–2560. 10.1111/sms.14482 37642310

[CR7] Clarkson PM, Hubal MJ (2002) Exercise-induced muscle damage in humans. Am J Phys Med Rehabil 81:S52–S69. 10.1097/00002060-200211001-00007 12409811

[CR8] del Vecchio A, Negro F, Felici F, Farina D (2017) Associations between motor unit action potential parameters and surface EMG features. J Appl Physiol 123:835–843. 10.1152/japplphysiol.00482.2017 28751374

[CR9] Enoka RM, Duchateau J (2008) Muscle fatigue: what, why and how it influences muscle function. J Physiol 586:11–23. 10.1113/jphysiol.2007.139477 PMC2375565 17702815

[CR10] Exercise & Sports Science Australia (2019) Adult pre-exercise screening system (APSS): Version 2. Brisbane, QLD: Exercise and Sports Science Australia. https://www.essa.org.au/Common/Uploaded%20files/Tools%20and%20templates/Adult%20Pre%20Screening%20Tool%202021.pdf. Accessed 9 June 2026

[CR11] Farina D, Holobar A, Merletti R, Enoka RM (2010) Decoding the neural drive to muscles from the surface electromyogram. Clin Neurophysiol 121:1616–1623. 10.1016/j.clinph.2009.10.040 20444646

[CR12] Faul F, Erdfelder E, Buchner A, Lang A-G (2009) Statistical power analyses using G*Power 3.1: tests for correlation and regression analyses. Behav Res Methods 41:1149–1160. 10.3758/brm.41.4.1149 19897823

[CR13] Hahn D, Hoffman BW, Carroll TJ, Cresswell AG (2012) Cortical and spinal excitability during and after lengthening contractions of the human plantar flexor muscles performed with maximal voluntary effort. PLoS ONE 7:e49907. 10.1371/journal.pone.0049907 PMC3498207 23166794

[CR14] Halekoh U, Højsgaard S (2014) A Kenward-Roger approximation and parametric bootstrap methods for tests in linear mixed models: the R package pbkrtest. J Stat Softw 59(9):1–30. 10.18637/jss.v059.i09

[CR15] Henschke J, Zecher MM, Mayer F, Engel T (2022) Contralateral repeated bout effect following preconditioning exercises: a systematic review. Sport Sci Health 18:1–10. 10.1007/s11332-021-00804-0

[CR16] Hermens HJ, Freriks B, Disselhorst-Klug C, Rau G (2000) Development of recommendations for SEMG sensors and sensor placement procedures. J Electromyogr Kinesiol 10:361–374. 10.1016/s1050-6411(00)00027-4 11018445

[CR17] Hody S, Croisier J-L, Bury T, Rogister B, Leprince P (2019) Eccentric muscle contractions: risks and benefits. Front Physiol 10:536. 10.3389/fphys.2019.00536 PMC6510035 31130877

[CR18] Hoffman BW, Cresswell AG, Carroll TJ, Lichtwark GA (2014) Muscle fascicle strains in human gastrocnemius during backward downhill walking. J Appl Physiol 116:1455–1462. 10.1152/japplphysiolW.01431.2012 23558392

[CR19] Hoffman BW, Cresswell AG, Carroll TJ, Lichtwark GA (2016) Protection from muscle damage in the absence of changes in muscle mechanical behavior. Med Sci Sports Exerc 48:1495–1505. 10.1249/MSS.0000000000000920 27433958

[CR20] Howatson G, van Someren K (2007) Evidence of a contralateral repeated bout effect after maximal eccentric contractions. Eur J Appl Physiol 101:207–214. 10.1007/s00421-007-0489-5 17534644

[CR21] Hyldahl RD, Chen TC, Nosaka K (2017) Mechanisms and mediators of the skeletal muscle repeated bout effect. Exerc Sport Sci Rev 45:24–33. 10.1249/jes.0000000000000095 27782911

[CR22] Ishikawa M, Komi PV, Grey MJ, Lepola V, Bruggemann GP (2005) Muscle-tendon interaction and elastic energy usage in human walking. J Appl Physiol 99:603–608. 10.1152/japplphysiol.00189.2005 15845776

[CR23] Jeon S, Kang M, Ye X (2023) Contralateral protective effect against repeated bout of damaging exercise: a meta-analysis. Res Sports Med 31:137–156. 10.1080/15438627.2021.1954512 34304646

[CR24] Kenward MG, Roger JH (1997) Small sample inference for fixed effects from restricted maximum likelihood. Biometrics 53:983–997. 10.2307/2533558 9333350

[CR25] Kuznetsova A, Brockhoff PB, Christensen RHB (2017) lmerTest package: tests in linear mixed effects models. J Stat Softw 82:1–26. 10.18637/jss.v082.i13

[CR26] Lenth RV, Piaskowski J (2026) emmeans: estimated marginal means, aka least-squares means (R package version 2.0.2). 10.32614/CRAN.package.emmeans

[CR27] Lichtwark GA, Wilson AM (2007) Is Achilles tendon compliance optimised for maximum muscle efficiency during locomotion? J Biomech 40:1768–1775. 10.1016/j.jbiomech.2006.07.025 17101140

[CR28] Locher C, Koechlin H (2022) Do expectations influence pain? Recognizing Irving Kirsch’s contribution to our understanding of pain. Am J Clin Hypn 65:211–222. 10.1080/00029157.2022.2121677 36219555

[CR29] Marathamuthu S, Selvanayagam VS, Yusof A (2022) Contralateral effects of eccentric exercise and DOMS of the plantar flexors: evidence of central involvement. Res Q Exerc Sport 93:240–249. 10.1080/02701367.2020.1819526 32976088

[CR30] McHugh MP (2003) Recent advances in the understanding of the repeated bout effect: the protective effect against muscle damage from a single bout of eccentric exercise. Scand J Med Sci Sports 13:88–97. 10.1034/j.1600-0838.2003.02477.x 12641640

[CR31] McNulty KL, Elliott-Sale KJ, Dolan E, Swinton PA, Ansdell P, Goodall S, Thomas K, Hicks KM (2020) The effects of menstrual cycle phase on exercise performance in eumenorrheic women: a systematic review and meta-analysis. Sports Med 50:1813–1827. 10.1007/s40279-020-01319-3 PMC7497427 32661839

[CR32] Morgan DL (1990) New insights into the behavior of muscle during active lengthening. Biophys J 57:209–221. 10.1016/s0006-3495(90)82524-8 PMC1280663 2317547

[CR33] National Health and Medical Research Council (2023) National Statement on Ethical Conduct in Human Research 2023. National Health and Medical Research Council. https://www.nhmrc.gov.au/about-us/publications/national-statement-ethical-conduct-human-research-2023. Accessed 9 June 2026.

[CR34] Nosaka K, Sakamoto K, Newton M, Sacco P (2001) The repeated bout effect of reduced-load eccentric exercise on elbow flexor muscle damage. Eur J Appl Physiol 85:34–40. 10.1007/s004210100430 11513318

[CR35] Paschalis V, Margaritelis NV, Chatzinikolaou PN, Theodorou AA, Nikolaidis MG (2026) Eccentric exercise and muscle damage: an introductory guide. J Funct Morphol Kinesiol 11:139. 10.3390/jfmk11020139 PMC13108084 42029507

[CR36] Pincheira PA, Lichtwark GA, Brown NAT, Carroll TJ, Cresswell AG, Hoffman BW (2018) The repeated bout effect can occur without mechanical and neuromuscular changes after a bout of eccentric exercise. Scand J Med Sci Sports 28:2123–2134. 10.1111/sms.13222 29790207

[CR37] Pincheira PA, Hoffman BW, Cresswell AG, Carroll TJ, Brown NAT, Lichtwark GA (2021a) Cyclic eccentric stretching induces more damage and improved subsequent protection than stretched isometric contractions in the lower limb. Eur J Appl Physiol 121:3349–3360. 10.1007/s00421-021-04787-1 34436674

[CR38] Pincheira PA, Martinez-Valdes E, Guzmán-Venegas R, Falla D, Garrido MI, Cresswell GA, Lichtwark G (2021b) Regional changes in muscle activity do not underlie the repeated bout effect in the human gastrocnemius muscle. Scand J Med Sci Sports 31:799–812. 10.1111/sms.13912 33378553

[CR39] Pincheira PA, Mayfield DL, Fox AS, Brown NA, Carroll TJ, Cresswell AG, Lichtwark GA (2023) Increased muscle force does not induce greater stretch-induced damage to calf muscles during work-matched heel drop exercise. Eur J Appl Physiol 123:1801–1808. 10.1007/s00421-023-05188-2 PMC10363059 37055644

[CR40] R Core Team (2026) *R: A language and environment for statistical computing* (Version 4.5.3). R Foundation for Statistical Computing, Vienna

[CR41] Starbuck C, Eston RG (2012) Exercise-induced muscle damage and the repeated bout effect: evidence for cross transfer. Eur J Appl Physiol 112:1005–1013. 10.1007/s00421-011-2053-6 21720885

[CR42] Stožer A, Vodopivc P, Bombek LK (2020) Pathophysiology of exercise-induced muscle damage and its structural, functional, metabolic, and clinical consequences. Physiol Res 69:565–598. 10.33549/physiolres.934371 PMC8549894 32672048

[CR43] Tsuchiya Y, Nakazato K, Ochi E (2018) Contralateral repeated bout effect after eccentric exercise on muscular activation. Eur J Appl Physiol 118:1997–2005. 10.1007/s00421-018-3933-9 29987366

[CR44] World Medical Association (2013) World Medical Association Declaration of Helsinki: ethical principles for medical research involving human subjects. JAMA 310:2191–2194. 10.1001/jama.2013.281053 24141714

[CR45] Xin L, Hyldahl R, Chipkin S, Clarkson P (2014) A contralateral repeated bout effect attenuates induction of NF-κB binding following eccentric exercise. J Appl Physiol 116:1473–1480. 10.1152/japplphysiol.00133.2013 23950163

