# Supplementary material for: Contralateral repeated bout effect in the triceps surae after unilateral heel drop exercise at 2- and 7-day interbout intervals
Source: Eur J Appl Physiol. 2026 Sep 8;126(11):6449–64. doi: 10.1007/s00421-026-06421-4 (PMC13751724; doi:10.1007/s00421-026-06421-4)
Supplement: Supplementary file 1 [file 421_2026_6421_MOESM1_ESM.pdf]

Article title: Contralateral Repeated Bout Effect in the Triceps Surae After Unilateral Heel Drop Exercise at 2- and 7-Day Interbout Intervals

Journal: European Journal of Applied Physiology

Authors:

Nicole S. Jones[1,2] (ORCID: 0000-0002-6745-2568)

Dean E. Mills[1,2] (ORCID: 0000-0002-2653-169X)

Patricio A. Pincheira[1,2,3] (ORCID: 0000-0001-8528-1515)

Ben W. Hoffman[1,2] (ORCID: 0000-0002-8408-6192)

Corresponding author: Nicole S. Jones, Nicole.Jones@unisq.edu.au

Affiliations:

[1] University of Southern Queensland, School of Health, Psychological, and Medical Sciences, Ipswich, Queensland, Australia.

[2] University of Southern Queensland, Institute for Health, Springfield, Queensland, Australia.

[3] University of Queensland, School of Human Movement and Nutrition Sciences, Brisbane, Queensland, Australia.

Online Resource 1. Exercise task characteristics during the first (ECC1) and second (ECC2) eccentric exercise bouts in the 2-day (2D) and 7-day (7D) interbout interval groups.

Eccentric Exercise Task Characteristics

The eccentric phase of each heel drop was identified from the ankle angle signal as the descending portion of the movement. The duration of this phase was defined as the time under tension. Impulse was calculated as the integral of the vGRF over the same period. Mechanical work was approximated as a within-participant work index from the synchronised vGRF and ankle angle data, computed as:

$$Work\ Index = mean\ vGRF \times Achilles\ tendon\ moment\ arm \times angular\ displacement$$

where angular displacement was expressed in radians.

A constant Achilles tendon moment arm of 0.05 m was applied to all participants as an order-of-magnitude estimate consistent with in vivo MRI-based adult values (approximately 52.5 ± 5.6 mm) (Alexander et al., 2017). Achilles tendon moment arm is known to vary with ankle angle and to increase during strong plantar flexion activation compared with rest (Maganaris et al., 1998). Accordingly, this work index was used only to assess the within-participant consistency of task execution across bouts and was not intended to quantify absolute musculotendinous work. Angular displacement was calculated for each participant as the mean ankle joint angular excursion during the eccentric phase, which was derived directly from the electrogoniometer signal. Mean values across all valid repetitions were obtained for each bout. These variables served as indicators of task execution consistency, rather than experimental outcomes.

Supplementary Table S1. Exercise task characteristics during the first (ECC1) and second (ECC2) eccentric bouts in the 2-day (2D) and 7-day (7D) interbout interval groups. Values are mean ± SD. The 95% confidence intervals (CI) represent the within-group standardised mean change (d_z) from ECC1 to ECC2 and are provided to aid interpretation of the bout-to-bout consistency in task execution.

Variable	Group	ECC1 Mean ± SD	ECC2 Mean ± SD	95% CI (standardised change)
**Total Time Under Tension (s/bout)**	2D	873 ± 136	832 ± 93	[−1.05, 0.24]
	7D	916 ± 46	904 ± 93	[−1.04, 0.41]
**Time Under Tension (s/rep)**	2D	3.84 ± 0.63	3.69 ± 0.43	[−1.04, 0.26]
	7D	4.12 ± 0.32	4.01 ± 0.36	[−1.57, 0.26]
**Range of Motion (°/rep)**	2D	51.7 ± 11.1	44.6 ± 15.7	[−1.74, −0.50]
	7D	46.9 ± 6.0	45.9 ± 4.8	[−0.80, 0.46]
**Vertical Ground Reaction Force (N/rep)**	2D	718 ± 149	761 ± 166	[0.29, 2.45]
	7D	713 ± 178	730 ± 192	[−0.38, 0.95]
**Impulse (N·s/rep)**	2D	2688 ± 473	2767 ± 514	[−0.44, 0.85]
	7D	2931 ± 845	2935 ± 864	[−0.87, 0.51]
**Estimated Work Index (J/bout)**	2D	7175 ± 1369	6469 ± 2323	[−1.1, 0.12]
	7D	6420 ± 1452	6515 ± 1426	[−0.45, 1.01]

References

Alexander C, Lum I, Reid S, Clarke E, Stannage K, El-Sallam Abd A, Herbert R, Donnelly C (2017) A simple but reliable method for measuring 3D Achilles tendon moment arm geometry from a single static magnetic resonance scan. J Biomech 55:134–138. https://doi.org/10.1016/j.jbiomech.2017.01.038

Maganaris CN, Baltzopoulos V, Sargeant AJ (1998) Changes in Achilles tendon moment arm from rest to maximum isometric plantarflexion: in vivo observations in man. J Physiol 510:977–985. https://doi.org/10.1111/j.1469-7793.1998.977bj.x
