# Supplementary material for: Contralateral repeated bout effect in the triceps surae after unilateral heel drop exercise at 2- and 7-day interbout intervals
Source: Eur J Appl Physiol. 2026 Sep 8;126(11):6449–64. doi: 10.1007/s00421-026-06421-4 (PMC13751724; doi:10.1007/s00421-026-06421-4)
Supplement: Supplementary file 2 [file 421_2026_6421_MOESM2_ESM.pdf]

Article title: Contralateral Repeated Bout Effect in the Triceps Surae After Unilateral Heel Drop Exercise at 2- and 7-Day Interbout Intervals

Journal: European Journal of Applied Physiology

Authors:

Nicole S. Jones[1,2] (ORCID: 0000-0002-6745-2568)

Dean E. Mills[1,2] (ORCID: 0000-0002-2653-169X)

Patricio A. Pincheira[1,2,3] (ORCID: 0000-0001-8528-1515)

Ben W. Hoffman[1,2] (ORCID: 0000-0002-8408-6192)

Corresponding author: Nicole S. Jones, Nicole.Jones@unisq.edu.au

Affiliations:

[1] University of Southern Queensland, School of Health, Psychological, and Medical Sciences, Ipswich, Queensland, Australia.

[2] University of Southern Queensland, Institute for Health, Springfield, Queensland, Australia.

[3] University of Queensland, School of Human Movement and Nutrition Sciences, Brisbane, Queensland, Australia.

Online Resource 2. Individual participant surface electromyography (sEMG) data corresponding to Fig. 4.

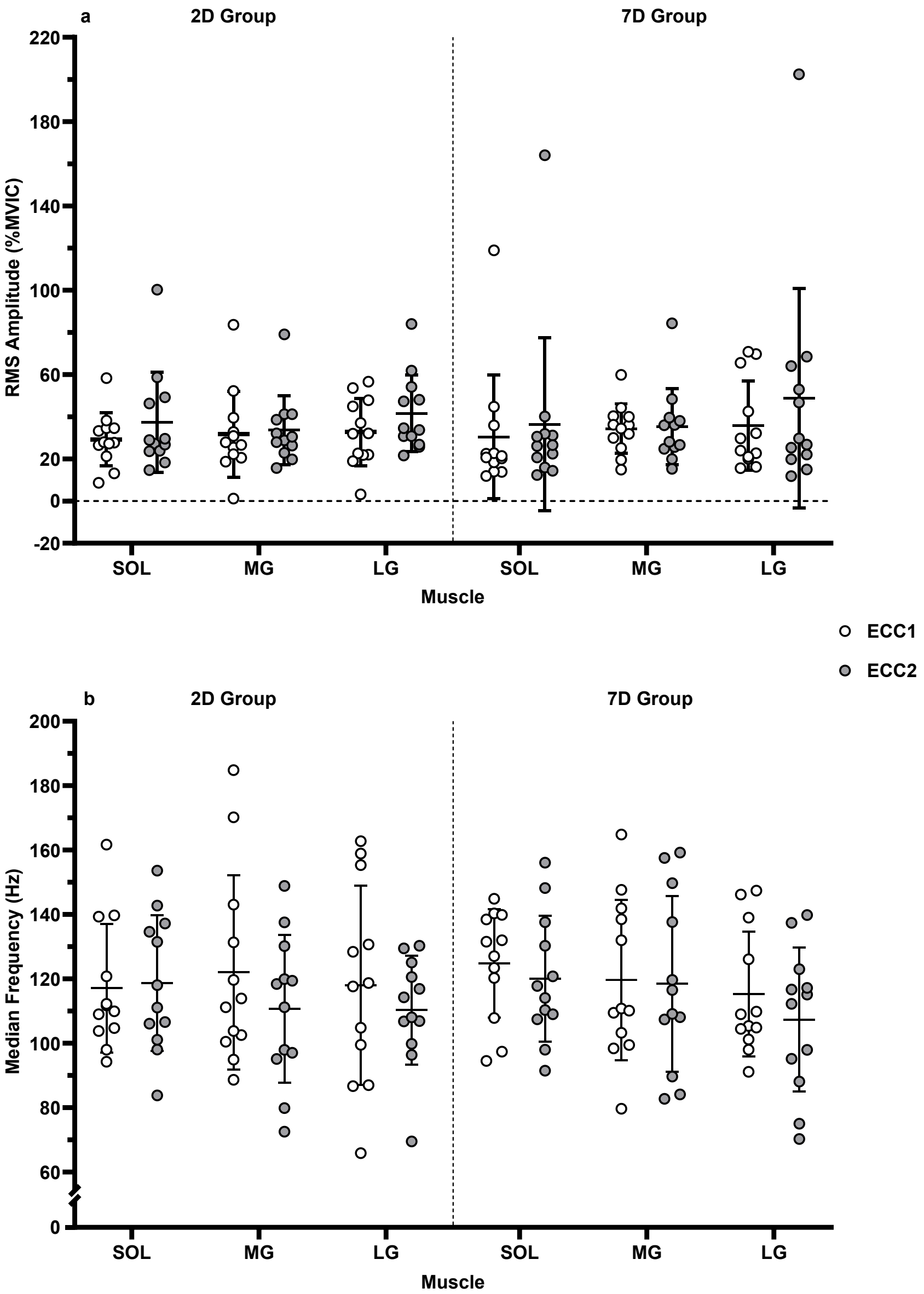

Supplementary Fig. S1. Individual participant surface electromyography (sEMG) data corresponding to Fig. 4. Panel **a** shows normalised root mean square (RMS) amplitude (% maximum voluntary isometric contraction; MVIC), and panel **b** shows median frequency (MDF; Hz), for the soleus (SOL), medial gastrocnemius (MG), and lateral gastrocnemius (LG) during the first (ECC1, open circles) and second (ECC2, grey circles) eccentric exercise bouts in the 2-day (2D) and 7-day (7D) interbout interval groups. Individual participant values are shown

with group means ± SD. RMS amplitude and MDF values were averaged across sets 3, 8, and 12.
