# Supplementary material for: Contralateral repeated bout effect in the triceps surae after unilateral heel drop exercise at 2- and 7-day interbout intervals
Source: Eur J Appl Physiol. 2026 Sep 8;126(11):6449–64. doi: 10.1007/s00421-026-06421-4 (PMC13751724; doi:10.1007/s00421-026-06421-4)
Supplement: Supplementary file 3 [file 421_2026_6421_MOESM3_ESM.pdf]

Article title: Contralateral Repeated Bout Effect in the Triceps Surae After Unilateral Heel Drop Exercise at 2- and 7-Day Interbout Intervals

Journal: European Journal of Applied Physiology

Authors:

Nicole S. Jones[1,2] (ORCID: 0000-0002-6745-2568)

Dean E. Mills[1,2] (ORCID: 0000-0002-2653-169X)

Patricio A. Pincheira[1,2,3] (ORCID: 0000-0001-8528-1515)

Ben W. Hoffman[1,2] (ORCID: 0000-0002-8408-6192)

Corresponding author: Nicole S. Jones, Nicole.Jones@unisq.edu.au

Affiliations:

[1] University of Southern Queensland, School of Health, Psychological, and Medical Sciences, Ipswich, Queensland, Australia.

[2] University of Southern Queensland, Institute for Health, Springfield, Queensland, Australia.

[3] University of Queensland, School of Human Movement and Nutrition Sciences, Brisbane, Queensland, Australia.

Online Resource 3. Reliability evidence for the plantar-flexor maximal voluntary isometric contraction (MVIC) protocol

The plantar-flexor MVIC protocol used in the present study was evaluated in a separate laboratory reliability dataset of 14 healthy adults (30 ± 7 years, 78 ± 14 kg, 1.77 ± 0.05 m; nine females and five males) using the same prone, heel-restrained, dorsiflexed testing configuration. Healthy adult participants completed repeated unilateral plantar-flexor MVIC assessments across within- and between-session comparisons, with torque recorded using the same dynamometry configuration and standardised strapping procedures. The protocol demonstrated excellent reliability and low measurement error, with intraclass correlation coefficients (ICC) of 0.93–0.98, coefficients of variation (CV) of 4.6–8.4%, standard errors of measurement (SEM) of 2–3 N·m, and 95% minimal detectable change (MDC95) values of 5–10 N·m. These reliability data support the use of this configuration for repeated plantar-flexor torque assessment and provide the measurement-error context used to interpret the absolute torque-loss contrasts in the present study.
